# Effect of Moderate-to-Severe Iodine Deficiency in Early Pregnancy on Subclinical Hypothyroidism: A Longitudinal Study in an Iodine-Sufficient Region in China

**DOI:** 10.3389/fnut.2022.839651

**Published:** 2022-04-01

**Authors:** Taotao Wang, Yanqiu Liu, Qianqian Kong, Xiaoxia Cao, Yuzhou Liu, Song Xia, Tingting Zheng, Li Yu

**Affiliations:** ^1^Department of Endocrinology and Clinical Nutrition, Affiliated Hospital of Jiangsu University, Zhenjiang, China; ^2^Department of Obstetrics, Affiliated Hospital of Jiangsu University, Zhenjiang, China; ^3^Department of Radiology, Affiliated Hospital of Jiangsu University, Zhenjiang, China; ^4^Department of Nuclear Medicine, Affiliated Hospital of Jiangsu University, Zhenjiang, China

**Keywords:** early pregnancy, iodine deficiency, subclinical hypothyroidism, urinary iodine concentration (UIC), longitudinal studies

## Abstract

**Objective:**

To investigate iodine status among pregnant women in an iodine-sufficient region in China after the implementation of revised universal salt iodization (USI) standards in 2012 and assess the association between urinary iodine concentrations (UIC) in early pregnancy and the incidence of subclinical hypothyroidism (SCH) in euthyroid women negative for antithyroid Ab during different trimesters.

**Methods:**

We measured the iodine status of 1,264 pregnant women, and performed follow-up assessment of thyroid function at 20 and 30 weeks of gestation among a cohort of 250 euthyroid women. We assessed the association of UIC in the 1st trimester with the incidence of SCH in subsequent trimesters. UIC and serum free triiodothyronine (FT3), free thyroxine (FT4), thyroid-stimulating hormone (TSH), thyroid peroxidase antibody (TPOAb), and thyroglobulin antibody (TgAb) were measured.

**Results:**

The median UIC was 135.95 μg/L among 1,264 women. Serum FT4 level was significantly higher in the group of UIC 150 to 249 μg/L compared with other UIC groups (*P* < 0.001). TSH was significantly higher in the UIC more than or equal to 250 μg/L group than the UIC 150 to 249 g/L group (*P* = 0.043). Of the 250 euthyroid women negative for antithyroid Ab (TSH value of 2.5–3.55 mU/L) in the 1st trimester, pregnant women with UIC lower than 100 μg/L in the 1st trimester exhibited a significantly increased risk of SCH (odds ratio [OR] = 2.47; 95% confidence interval [CI] = 1.22–5.71; *P* = 0.012, according to the Chinese Medical Association criteria; OR = 5.22, 95% CI = 1.73–6.09, *P* = 0.004, according to ATA criteria) during the latter half of pregnancy compared with the UIC 150 to 249 μg/L group.

**Conclusion:**

Moderate-to-severe iodine deficiency (UIC lower than 100 μg/L) in the 1st trimester was associated with a significantly higher risk of SCH during the 2nd or 3rd trimesters among euthyroid pregnant women who had negative for antithyroid Ab. Women with SCH during pregnancy require regular UIC tests to maintain appropriate iodine status.

## Introduction

The most common thyroid disease during pregnancy is hypothyroidism, especially subclinical hypothyroidism (SCH). SCH is diagnosed by elevated serum thyroid-stimulating hormone (TSH) without elevated serum free thyroxine (FT4). A higher incidence can be observed in women who has a family history of thyroid disease, iodine insufficiency, and increasing age. Iodine insufficiency during pregnancy often leads to hypothyroidism and adverse pregnancy outcomes.

Iodine requirements are higher in pregnant women than in non-pregnant adults because of the increased fetal thyroid hormone requirements for brain development and increased maternal renal excretion (1). Previous studies have indicated that inappropriate iodine statues in pregnancy were potentially associated with a higher prevalence of thyroid dysfunction and negative effects on fetal neurological and cognitive development (2–6). Adequate iodine nutrition status is especially beneficial to the sufficient synthesis of thyroid hormones and the maintenance of normal thyroid function in the 1st trimester of pregnancy. In 2007, the World Health Organization (WHO)/UNICEF/IGD recommended criteria for iodine status assessment in pregnant women according to median urinary iodine concentrations (UICs); a median level of UIC of 150 to 249 μg/L denotes iodine sufficiency, a UIC of lower than 150 μg/L denotes iodine deficiency, and a UIC of more than or equal to 250 μg/L denotes iodine excess (7).

The Chinese Ministry of Health (MOH) released new standards for the iodine content in household iodized salt in 2012 (Chinese Standard GB 26878–2011). In the standard, the average level of iodine content in household iodized salt was lowered from 50 mg/kg to 25 mg/kg. In March 2012, Jiangsu Province complied with the regulations and allowed a range of 18–33 mg/kg for iodine content in salt. Implementation of the revised regulations may have resulted in increased iodine deficiency, especially in pregnant women. According to the latest national iodine nutrition survey, the urinary iodine level of the general population and children in Jiangsu Province belongs to the state of iodine sufficiency (8). Recently, an observational study conducted in northern Jiangsu Province assessed iodine status by the neonatal TSH and suggested an iodine deficiency in the region, in which the prevalence of neonatal TSH values > 5 mIU/L was 29.3% (9). However, UIC is currently the most practical biochemical marker of iodine nutrition according to the WHO and the 2017 American Thyroid Association (ATA) Guidelines (1). Now that 7 years have passed since the updated universal salt iodization (USI) standards in China, evaluating urinary iodine among pregnant women on the basis of appropriate indicators and screening thyroid function is a crucial task. The 2017 ATA Guidelines recommended raising the cutoff point for SCH from 2.5 to 4.0 mU/L TSH (1, 10). Cutoff values also depend on the measurement system used. The 2019 Chinese Medical Association (10) defined 3.55 mU/L TSH as the cutoff point for SCH when a Beckman Coulter measurement system is used. According to the guidelines, the assessment frequency for thyroid function throughout pregnancy depends on the level of thyroid-related hormones and antibodies at the first prenatal visit and the diagnostic criteria for thyroid diseases during pregnancy. Therefore, the guidelines recommended that thyroid function and UIC monitoring were not necessary for women negative for thyroid peroxidase antibody (TPOAb) with a TSH value of 2.5–3.55 mU/L at their first prenatal visit. However, one study reported that the prevalence of SCH in subsequent trimesters was not lower for such women (11), and ultimately the new guidelines may result in a missed diagnosis of SCH. Thyroid hormones are necessary throughout pregnancy, and normal maternal thyroid function in the 2nd and 3rd trimesters of pregnancy remains crucial for fetal nervous system development. SCH has been associated with negative pregnancy outcomes and neurocognitive deficits in fetal development, even in women negative for antithyroid Ab (12, 13). Therefore, euthyroid pregnant women with a TSH between the cutoff values of the old and new SCH criteria (TSH value of 2.5–3.55 mU/L) should be monitored for SCH. Identifying a simple, non-invasive index of SCH risk for such groups should be a priority.

The aim of this study was two-fold: first, to assess the iodine status of pregnant women 7 years after the implementation of the adjusted USI standards in Jiangsu, China; second, to conduct a longitudinal study on euthyroid pregnant women who were negative for antithyroid Ab (TSH value of 2.5–3.55 mU/L) to identify an association between maternal iodine status in the 1st trimester of pregnancy and the risk of SCH in subsequent trimesters of pregnancy in an iodine-sufficient region.

## Methods

### Participants and Data Collection

A total of 1,264 pregnant women with a single pregnancy in the Affiliated Hospital of Jiangsu University (Zhenjiang, Jiangsu, China) from January 2019 to December 2019 were included in this study, and the area map for the sampling territory was shown in Supplementary Figure 1. All participants were aged 19–43 years and had lived in Jiangsu Province for more than 1 year. Women with a history or family history of thyroid diseases or chronic diseases, (e.g., hypertension, diabetes, liver insufficiency, and renal insufficiency and so on), or who had taken an iodine-containing multivitamin or iodinated contrast agents before pregnancy were excluded. Demographic characteristics including age, education level, ethnicity, gestational weeks, height, weight, body mass index (BMI) and medical and supplement-taking histories were collected by a questionnaire. In the first part of the cross-sectional study, 1,264 pregnant women were divided into three groups: 1st trimester (4–12 weeks), 2nd trimester (13–27 weeks), and 3rd trimester (≥ 28 weeks). UIC frequency distributions were assessed according to the WHO criteria: moderate-to-severe iodine deficiency (UIC lower than 100 μg/L), mild iodine deficiency (UIC 100 to 149 μg/L), adequate iodine (UIC 150 to 249 μg/L), above requirements (UIC 250 to 499 μg/L), and excessive (UIC more than or equal to 500 μg/L). This study was approved by the Medical Ethics Committee of the Affiliated Hospital of Jiangsu University (Approval # K0122).

### Sample Collection and Laboratory Measurements

**Figure 1:**
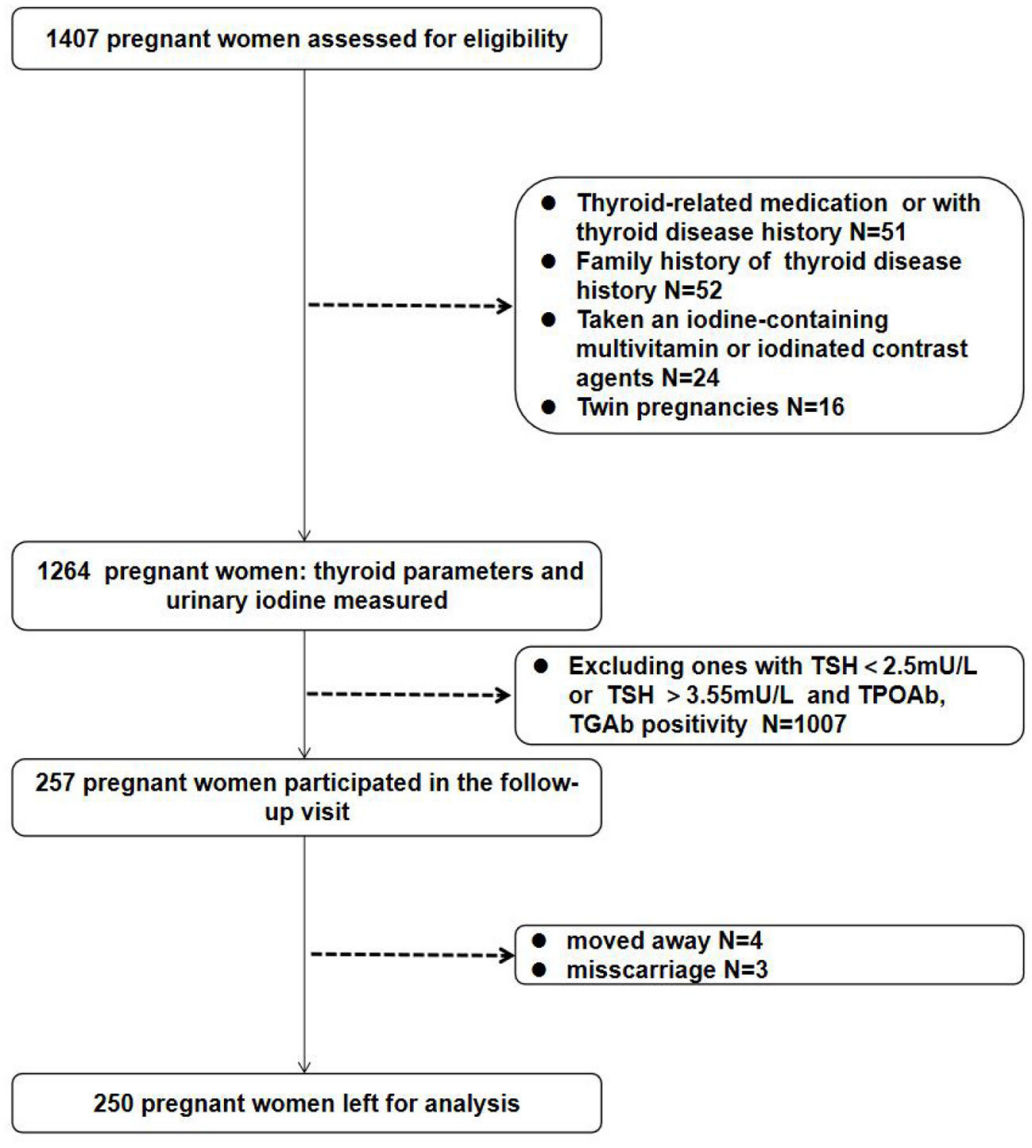
Participant selection.

Overnight fasting blood samples were obtained in evacuated tubes for blood specimen collection (4–5 mL), serum was separated and stored in the eppendorf tube at −80 °C for testing serum concentrations of free triiodothyronine (FT3), FT4, TSH, TPOAb, and thyroglobulin antibody (TGAb). Thyroid hormones and antibodies were measured using chemiluminescent detection on a Beckman Coulter Unicel DxI 800 (Beckman Coulter Inc., Miami, FL, USA) in the Nuclear Medicine Laboratory of our hospital. The intra-assay coefficients of variation of serum FT3, FT4, TSH, TPOAb, and TgAb were 1.47–4.64%, 1.80–5.01%, 1.31–3.79%, 2.35–4.23%, and 1.66–4.21%, respectively. The inter-assay coefficients of variation of serum FT3, FT4, TSH, TPOAb and TgAb were 2.02–5.13%, 2.42–6.57%, 1.35–4.71%, 4.03–5.16%, and 2.54–5.78%, respectively. Spot urine samples were collected 3 days after the first prenatal visit to ensure that pregnant women did not consume any iodine-rich foods (e.g., seaweed, kelp, and seafood) within 3 days of the sampling. Subsequently, further spot urinary iodine samples were collected twice after the first prenatal visit; three samples were collected in all subsequent weeks, and the mean value of the three urinary iodine tests was used as the final urinary iodine value to reduce the effect of glomerular filtration on measured UIC. Participants determined to be iodine deficient after the UIC spot-tests were recommended an iodine-rich diet but not iodine-containing multivitamins or drugs; likewise, a decrease in iodine-rich food was suggested to participants with excessive iodine. The urine samples (10 mL) were stored in plastic bottles and stored at 4 °C until analysis. Of the 1,264 participants, follow-up visits at 20 and 30 weeks of gestation were performed for 257 participants who were euthyroid and antithyroid Ab-negative with a TSH value of 2.5–3.55 mU/L in the 1st trimester. Four participants were lost to follow-up, and three pregnancies ended in miscarriage. Therefore, 250 women completed the longitudinal study. Blood samples were obtained at the first visit and at 20 and 30 weeks of gestation (Figure 1). UIC was evaluated using As^3+^-Ce^4+^ catalytic spectrophotometry on a iodine meter (OTT-I-P, Xiangyang Wentes Health Technology Co., Ltd, Hubei, China) according to a health industry standard method released by the National Health Commission of China in 2016 (WS/T 107.1–2016) in the Nuclear Medicine Laboratory of our hospital, in which freeze-dried human urinary iodine component obtained from National Institute for Nutrition and Health Chinese Center for Disease Control and Prevention is used as the certified reference material.

### Diagnosis of Thyroid Disease

The evaluation of thyroid dysfunction was conducted using different reference intervals, one of which was recommended by the 2019 Chinese Medical Association guidelines (14): T1: FT4 9.01–15.89 pmol/L,TSH 0.05–3.55 mU/L, T2: FT4 6.62–13.51 pmol/L„TSH 0.21–3.31 mU/L; T3: FT4 6.42–10.75 pmol/L, TSH 0.43–3.71 mU/L; TPOAb 0–9 IU/mL, and TGAb 0–4 IU/mL for three trimestres. A cutpoint of 4.0 mU/L for TSH proposed by the ATA in 2017 (1) was also used. Diagnosis of thyroid disease: Clinical hypothyroidism: TSH is higher than the normal range with FT4 lower than normal range; SCH: TSH is higher than the reference range and FT4 within the reference values; isolated hypothyroxinemia: TSH within the reference values and FT4 lower than the normal range as well as negative TPOAb and negative TgAb. Positive TPOAb: TPOAb > 9 IU/mL. Positive TGAb: TGAb > 4 IU/mL.

### Statistical Analysis

Statistical analysis was performed using SPSS 16.0 (SPSS Inc., Chicago, IL, USA). Variables were presented as mean ± standard deviation or medians with interquartile ranges. One-way analysis of variance was used to compared continous variables, Kruskal-Wallis method was used to compared non-normally distributed data, Dunn's test with Bonferroni correction was used for pairwise multiple comparision. Categorical variables were compared using the chi-square test and were expressed as counts and percentages. Multivariate analyses were used to estimate the association between iodine deficiency and SCH during different trimesters. All the tests were two-tailed and values of *P* < 0.05 were considered statistically significant.

## Results

### Participant Characteristics

Table 1 shows the demographic characteristics, UICs, serum FT3, FT4, TSH, and UIC frequency distribution according to pregnancy trimesters. A total of 987 (78.09%), 169 (13.37%), and 108 (8.54%) participants were in the 1st, 2nd and 3rd trimesters of pregnancy, respectively. The average age was 28.36 ± 3.64 (range: 18–43) years. The average BMI was 21.69 ± 2.05 kg/m^2^. Age, ethnicity, educational level, and pre-pregnancy BMI did not significantly differ between the three trimesters of pregnancy.

**Table 1:** Demographic characteristics and urinary iodine concentration frequency distribution based on pregnant trimesters.

**Variable**	**First trimester**	**Second trimester**	**Third trimester**	***P* Value**
Number of women	987	169	108	*-*
Age, year	28.38 ±4.11	28.88 ±4.43	28.07 ± 2.52	0.154
Gestational weeks, week	9 (7–11)	18 (16–20)	30 (28–34)	<0.001
Ethnicity, n (%)				
Han majority	984 (99.70)	168 (99.41)	107 (99.1)	0.302
Other minority	3 (0.30)	1 (6.13)	1 (0.93)	0.302
Education, n (%)				
Elementary school or no education	101 (10.23)	18 (10.65)	12 (11.11)	0.952
Middle or high school	177 (17.93)	28 (16.57)	19 (17.59)	0.911
College or above	709 (71.83)	123 (72.78)	77 (71.29)	0.958
Weight, kg	54.97 ± 6.63	54.11 ± 6.83	54.05 ± 5.17	0.781
BMI, kg/m^2^	22.04 ± 2.37	21.59 ± 2.22	21.45 ± 1.70	0.728
FT3, pmol/L				
Median	4.93	4.99	5.01	0.380
IQR	4.47–5.31	4.64–5.25	4.73–5.22	
FT4, pmol/L				
Median	12.15^**^	9.88^**^	9.36^**^	<0.001
IQR	10.23–16.02	8.83–11.57	8.02–10.48	
TSH, μIU/ml				
Median	1.66	1.68	1.32	0.090
IQR	1.11–2.49	1.12–2.31	0.83–2.02	
UIC, μg/L				
Median	147.1^*^	122.1^*^	110.6	0.030
IQR	93.1–184.8	89.25–167.7	84.3–170.7	
UIC frequency				
<50	50 (5.07)	9 (5.33)	6 (5.56)	0.970
50–99	225 (22.80)	49 (29.00)	33 (30.55)	0.063
100–149	226 (22.90)	50 (29.59)	27 (25.00)	0.160
150–249	364 (36.88)^#^	46 (27.22)^#^	31 (28.70)	0.019
250-499	91 (9.22)	11 (6.50)	9 (8.33)	0.730
≥500	31 (3.14)	4 (2.37)	2 (1.85)	0.675

*Data are presented as mean ± SD for normally distributed variables and as median with interquartile range for non-normally distributed variables. Categorical variables were expressed as counts and percentages.*

*Multiple comparisons were determined by Kruskal-Wallis test, Dunn's test with Bonferroni correction was used for pairwise comparison.*

* *Compared between the first trimester and the second trimester groups, P < 0.05.*

** *Compared among the three trimesters, P < 0.001;*

# *Analyzed by chi-square test, compared between the first trimester and the second trimester groups.*

*Abbreviations: UIC, urinary iodine concentrations; FT3, Free triiodothyronoine; FT4, free thyroxine; TSH, thyrotropin*.

### Iodine Status

The overall median UIC was 135.95 μg/L, and the median UIC was 147.1 μg/L, 122.1 μg/L, and 110.6 μg/L in the 1st, 2nd, and 3rd trimesters, respectively, suggesting that pregnant women in this area were iodine deficient in all stages of pregnancy. Compared with participants in the 1st trimester, pregnant women in the 2nd trimester had a significantly lower UIC *(P* = 0.017). No significant differences were observed in UIC among the other groups. According to the WHO criteria, 441 participants (34.89%) were iodine sufficient (UIC 150 to 249 μg/L), 372 (29.43%) had moderate-to-severe iodine deficiency (UIC lower than 100 μg/L), 303 (23.97%) had mild iodine deficient (UIC 100 to 149 μg/L), 111 (8.78%) had above requirements of iodine intake(UIC 250 to 499 μg/L), and 37 (2.93%) were iodine excessive (UIC more than or equal to 500 μg/L). The prevalence of sufficient iodine in pregnant women in the 1st trimester was higher than those in the 2nd and 3rd trimesters (36.88% vs. 27.22% and 28.70%; *P* = 0.019).

### Baseline Thyroid Function

Serum FT4 was found significantly higher in participants in the 1st trimester than in the 2nd (12.15 vs. 9.88 pmol/L; *P* < 0.001) or 3rd trimester (12.15 vs. 9.36 pmol/L; *P* < 0.001). Likewise, serum FT4 was significantly higher in the 2nd trimester than in the 3^rd^ trimester (9.88 vs. 9.36 pmol/L; *P* < 0.001). No significant differences were observed between FT3 and TSH among the three trimesters of pregnancy (Table 1). Serum FT4 was found significantly lower in pregnant women with UIC lower than 100 μg/L (*P* < 0.001), UIC 100 to 149 μg/L (*P* < 0.001), and UIC more than or equal to 250 μg/L than those with UIC 150 to 249 μg (*P* < 0.001). By contrast, TSH showed significantly higher in women with UIC more than or equal to 250 μg/L compared with UIC 150 to 249 μg/L group (*P* = 0.043), but no significant differences in TSH values were observed among the other groups (Table 2).

**Table 2:** Serum concentrations of FT3, FT4 and TSH in the first trimester among women by UIC group.

**UIC(μg/L)**	**n**	**FT3 (pmol/L)**	***P* Value**	**FT4(pmol/L)**	***P* Value**	**TSH(mU/L)**	***P* Value**
<100	275	4.96 (4.59–5.41)	0.043	11.41 (10.25–14.06)	<0.001	1.55 (1.03–2.52)	0.355
100–149	226	4.92 (4.35–5.29)	0.761	11.39 (9.71–15.22)	<0.001	1.68 (1.17–2.53)	0.734
150–249	364	4.86 (4.43–5.24)	Ref	14.43 (10.79–17.32)	Ref	1.62 (1.02–2.36)	Ref
≥250	122	4.97 (4.50–5.33)	0.406	11.13 (9.94–14.98)	<0.001	1.74 (1.27–2.33)	0.043

*Data are presented as median with interquartile range.*

*Ref: reference category; P values represent the median level of this group compared with the reference group*.

### Prevalence of Thyroid Disorders

Table 3 lists the prevalence of thyroid diseases according to the Chinese Medical Association and ATA criteria. The lowest prevalence of TPOAb and TgAb positivity was in the UIC 150 to 249 μg/L group. The highest prevalence of SCH was in pregnant women with UIC more than or equal to 250 μg/L. The highest rate of clinical hypothyroidism, isolated hypothyroxinemia, and TPOAb positivity was in pregnant women with UIC lower than 100 μg/L, but the difference between groups was non-significant. Compared with the UIC 150 to 249 μg/L group, the prevalence of TgAb positivity was significantly higher in UIC lower than 100 μg/L group (12.37% vs. 6.12%, *P* = 0.002). The prevalence of SCH and clinical hypothyroidism was higher according to the Chinese Medical Association criteria than the ATA criteria (4.43% vs. 3.09%; *P* = 0.075, 1.27% vs. 0.64%; *P* = 0.101).

**Table 3:** Prevalence of thyroid disorders among women by UIC group.

	**UIC (μg/L)**					
	**<100**	**100–149**	**150–249**	**≥250**	**Total**	***P* Value**
Number of participants	372	303	441	148	1264	
Subclinical hypothyroidism, n(%)						
Chinese criteria	20 (5.38)	10 (3.30)	16 (3.63)	10 (6.75)	56 (4.43)	0.235
ATA criteria	13 (3.49)	7 (2.31)	11 (2.49)	8 (5.41)	39 (3.09)	0.262
Clinical Hypothyroidism, n(%)						
Chinese criteria	6 (1.61)	4 (1.32)	4 (0.91)	2 (1.35)	16 (1.27)	0.838
ATA criteria	4 (1.08)	1 (0.33)	2 (0.45)	1(0.68)	8 (0.64)	0.609
Isolated hypothyroxinemia, n(%)						
Chinese criteria	20 (5.38)	6 (1.98)	10 (2.27)	5 (3.38)	41 (3.24)	0.050
ATA criteria	22 (5.91)	9 (2.97)	12 (2.72)	6 (4.05)	49 (3.88)	0.094
TPOAb positivity, n(%)	43 (11.56)	27 (8.91)	30 (6.80)	16 (10.81)	116 (9.18)	0.111
TGAb positivity, n(%)	46 (12.37)	29 (9.57)	27 (6.12)	17 (11.49)	119 (9.41)	0.017

*Data are presented as n (%), analyzed by chi-square test, comparisons of prevalence of thyroid disorders between diffenent UIC groups.*

*Thyroid disorders are diagnosed based on different TSH upper reference limits, Chinese criteria (criteria by Chinese Medical Association): TSH upper reference limit of 3.55 mU/L; ATA criteria (criteria by American Thyroid Association): TSH upper reference limit of 4.0 mU/L*.

### Prevalence of SCH in the 2nd and 3rd Trimesters

To assess the association between UIC in the 1st trimester and the incidence of SCH in the 2nd and 3rd trimesters, follow-up visits were performed at 20 and 30 weeks of gestation with 250 women who had normal thyroid function (TSH 2.5–3.55 mU/L) and negative TPOAb and TgAb tests in the 1st trimester. Table 4 presents the baseline characteristics and thyroid function in the 1st trimester and in follow-up visits. No significant differences in age, gestational weeks, pre-pregnancy BMI or serum concentrations of FT3, FT4 or TSH in the 1st trimester were observed across UIC groups.

**Table 4:** Baseline characteristics and thyroid function in the first trimester among follow-up visits of pregnant women by UIC group.

**Parameters**	**UIC in the first trimester, μg/L**				***P* Value**
	**<100 (*n* = 47)**	**100–149 (*n* = 72)**	**150–249 (*n* = 94)**	**≥250 (*n* = 37)**	
Age, year	28.44 ± 3.82	28.71 ± 3.78	29.01 ± 4.63	27.65 ± 3.73	0.511
Gestational age, weeks	10 (8–12)	9 (8–11)	10 (7–12)	9 (8–12)	0.322
Pre-pregnancy Weight, kg	54.60 ± 4.17	54.23 ± 4.32	54.72 ± 3.99	54.48 ± 3.92	0.467
Pre-pregnancy BMI, kg/m^2^	22.57 ± 2.11	22.45 ± 2.06	22.29 ± 2.18	22.04 ± 1.39	0.721
Thyroid function in first trimester					
FT3, pmol/L	4.82 (4.62–5.31)	4.87 (4.30–5.19)	5.04 (4.56–5.27)	5.02 (4.49–5.11)	0.815
FT4, pmol/L	11.02 (9.18–12.62)	10.58 (9.37–15.16)	12.42 (10.66–5.21)	10.39 (9.78–12.31)	0.355
TSH, mU/L	2.95 (2.70–3.22)	2.97 (2.76–3.35)	2.84 (2.66–3.14)	2.80 (2.82–3.17)	0.373

*Data are presented as median with interquartile range for non-normally distributed variables.*

*Multiple comparisons were determined by Kruskal-Wallis test*.

Of the 250 euthyroid pregnant women negative for antithyroid Ab (TSH 2.5–3.55 mU/L) in the 1st trimester, the incidence of SCH in the 2nd or 3rd trimesters according to different diagnostic criteria is presented in Table 5. According to the Chinese Medical Association criteria, 56 (22.40%) participants were confirmed to have SCH in the 2nd or 3rd trimesters, whereas according to the ATA criteria, 29 (11.60%) participants were confirmed to have SCH (TSH ≥ 4.00 mU/L). If the ATA 2012 criteria were used (TSH ≥ 3.0 mU/L), 131 (52.4%) participants would be considered as having SCH. The lowest prevalence of SCH in the 2nd or 3rd trimester was observed in the UIC 150 to 249 μg/L group (15.96% according to the Chinese Medical Association criteria and 5.32% according to the ATA criteria). We performed multiple logistic regression analyses to evaluate the association between UIC and SCH, and found that UIC lower than 100 μg/L in early pregnancy exhibited an increased risk of SCH (odds ratio [OR] = 2.47, 95% confidence interval [CI] = 1.22–5.71; *P* = 0.012, according to the Chinese Medical Association criteria; and OR = 5.22, 95% CI = 1.73–6.09; *P* = 0.004, according to the ATA criteria) in late pregnancy than those in the iodine sufficient group after adjusting for age, gestational age, and BMI.

**Table 5:** Multivariable logistic regression analyses of association between urinary iodine concentrations in the first trimester and incidence of subclinical hypothyroidism in second and third trimesters among pregnant women by Chinese and ATA diagnostic criteria.

**UIC in the first trimester (μg/L)**	**n**	**SCH by Chinese criteria (n,%)**	***P* Value**	OR (95%CI)^*^	**SCH by ATA criteria (n,%)**	***P* Value**	OR (95%CI)^*^
<100	47	20 (42.55)	0.012	2.47 (1.22–5.71)	11 (23.40)	0.004	5.22 (1.73–6.09)
100–149	72	12 (16.67)	0.897	1.02 (0.44–2.45)	8 (11.11)	0.168	1.85 (0.56–6.16)
150–249	94	15 (15.96)	Ref	Ref	5 (5.32)	Ref	Ref
≥250	37	9 (24.32)	0.875	1.50 (0.59–3.92)	5 (13.51)	0.114	2.72 (0.71–5.24)

Data are presented as n (%), ^*^Adjusted by age, gestational age and BMI. ^*^Analyzed by chi-square test, P < 0.05, ^*^compared with the UIC 150–249 μg/L group.

*Abbreviations: UIC, urinary iodine concentrations; SCH, subclinical hypothyroidism*.

## Discussion

According to the WHO (7) and 2017 ATA Guidelines (1), pregnant women are recommended to ingest approximately 250 μg of iodine daily to meet the requirements for fetal neurocognitive development and reduce the risk of thyroid dysfunction.

UIC is currently the most practical biochemical marker for iodine nutrition according to the WHO and ATA (7). UIC can be conveniently measured from a single spot urine sample, and such samples are commonly used in field research. However, single spot urine samples have reduced value for assessing iodine status during pregnancy because of the substantial increases in renal iodine and urine volume as the pregnancy progresses, resulting in variation in day-to-day spot urinary iodine values (1). In this study, three spot urinary iodine samples were collected every week beginning from 3 days after the first prenatal visit, and the median of the three urinary iodine values was used as the final urinary iodine value; the goal of this approach was to reduce the effect of glomerular filtration on measured UIC values.

In, 2012, China reduced the USI standards to prevent adverse effects from excessive iodine excess intake (8). A previous population-based study in Zhenjiang, Jiangsu Province in 2016 reported that the median UIC of pregnant women and school-age children was 161.5 and 225 μg/L, respectively, indicating that Zhenjiang residents had sufficient iodine intake (15). However, the rate of iodine deficiency in the participants in our study (as defined by the WHO; UIC lower than 150 μg/L) was 53.40%. The overall mean UIC determined in this study (135.95 μg/L) in pregnant women is similar to that reported in Zhejiang Province (130.47 μg/L) (16). The median UIC was the highest in the 1st trimester and the lowest in the 3rd trimester; these findings are consistent with studies in Shanghai and Switzerland (16, 17). The neglect of assessing iodine status and the lowering of iodine content in salt may be the causes of the iodine deficiency observed in this study. The fetal thyroid can begin to function and initiate the synthesis of thyroid hormones required for brain development with the support of maternal iodine transfer in the middle phase of gestation (18, 19). Therefore, pregnant women in the 2nd and 3rd trimesters should consider taking iodine supplements to ensure a normal iodine status.

However, no universally agreed upon diagnostic criteria exist for thyroid diseases during pregnancy because of the diversity in geography, ethnicity, trimester timing, and measurement systems. In 2017, the ATA raised the TSH cutoff point for SCH from 2.5 to 4.0 mU/L (3). The 2019 Chinese Medical Association guidelines recommended separate, specific reference ranges of pregnancy for thyroid function index based on several of the most widely used measurement systems in China (Abbott Laboratories, Roche Diagnostics, and Beckman Coulter) (14). In this study, a Beckman Coulter measurement system was used to assess serum thyroid functional parameters; therefore, a TSH cutoff of 3.55 mU/L was applied as the reference range.

Previous studies have indicated that both iodine deficiency and iodine excess during pregnancy can increase the risk of thyroid dysfunction (2–6). Our study revealed that the level of serum FT4 concentration was lower in the UIC more than or equal to 250 μg/L group than the iodine sufficient group. TSH was significantly higher in the UIC more than or equal to 250 μg/L group compared with the iodine sufficient group. Moreover, the prevalence of TPOAb positivity and SCH tended to be higher in the UIC more than or equal to 250 μg/L group compared with the iodine sufficient group, although the difference was non-significant, suggesting that thyroid autoimmunity may damage thyroid function due to increased iodination of thyroglobulin or direct stimulation of immune cells (20). This result is similar to that of Shi et al. (21) who reported a U-shaped relationship between UIC and the prevalence of TPOAb positivity. UIC more than or equal to 250 μg/L was identified to be associated with an increased risk of SCH (21).

Fetal and infant neurodevelopment depend on adequate thyroid hormone (22), and the increasing requirement for iodine during pregnancy makes pregnant women prone to iodine deficiency. In this study, FT4 was significantly lower in UIC 100 to 149 μg/L and UIC lower than 100 μg/L than that in the iodine sufficient group, indicating that sufficient iodine status is beneficial to achieving normal thyroid hormone levels. With the implementation of USI projects all over the world, severe iodine deficiencies have been greatly reduced (1). However, mild to moderate iodine deficiencies are still common during pregnancy (23). Mild iodine deficiencies have been reported in most European countries as well as the United States (24, 25). Similarly, iodine deficiencies have been widely reported in many regions of China since the implementation of the revised USI standards (15, 16, 26–30). Severe iodine deficiency (UIC lower than 50 μg/L) during pregnancy is associated with maternal and fetal thyroid dysfunction and destructive consequences on fetal intrauterine growth and neurological and cognitive development that can result in miscarriage, stillbirth, and infant mortality (31–33). Mild to moderate iodine deficiencies have also been associated with impaired cognitive outcomes in offsping (34–37), but not in all studies (38–40), The WHO recommends a daily iodine intake of approximately 250 μg during pregnancy. However, a randomized, double-blind, placebo-controlled trial (41) determined that iodine supplementation of 200 μg daily for women with UIC 131 μg/L in 1st trimenser had no benefit on offspring neurodevelopment in an iodine-sufficient area, in which mild iodine deficiency might have biased the above trial toward a null effect. Pregnant women whose TSH is between the cutoff values of the old and new ATA criteria (TSH value of 2.5–3.55) are likely to be identified as euthyroid and thyroid monitoring or even spot UIC measurements may not be a priority. In this study, most women (76%) with UIC lower than 100 μg/L in the 1st trimester of pregnancy remained at this level for the subsequent two tests, suggesting that iodine deficiency may be a long-term condition and increasing intake of iodine-rich food may not improve iodine status in the short term. Furthermore, we found that UIC lower than 100 μg/L in the 1st trimester of pregnancy is associated with a higher risk of SCH in subsequent stages of pregnancy. The underlying mechanisms may involve insufficient thyroid hormone synthesis and feedback stimulation of increased pituitary TSH production due to maternal iodine deficiency (42). Moreover, some cohort studies found that the offspring of pregnant women with UIC <100 μg/L showed adverse educational outcomes at school ages, which including lower intelligence quotient and reduced reading ability (36, 37). Future intervention trials in pregnant women with UIC <100 μg/L would be valuable.

The present study has several strengths, this is a longitudinal study, although the sample size was relatively small, the causal relationship between moderate-to-severe iodine deficiency in the first trimester of pregnancy and the increased risks of SCH in euthyroid women negative for antithyroid Ab during different trimesters could be convincing. In addition, our results identify a simple, non-invasive index of SCH risk during pregnancy. At the same time, the present study has several limitations that need to be addressed in future prospective studies. First, the number of participants in the final study was relatively small in part due to the strict inclusion criteria used. In particular, we excluded women who took an iodine-containing multivitamin before or during pregnancy, which was relatively common. Second, neonatal thyroid function and pregnancy outcomes were not observed in the follow-up analysis. Third, although UIC was determined on the basis of three spot urine samples to reduce the impact of glomerular filtration on the study results, we did not examine the urinary iodine-to-creatinine ratio, which has been suggested to be a valid index for correcting daily urinary volume for pregnant women (43). Fourth, the iodine intake from salt and other food of pregnant women wasn't evaluated according to a validated food-frequency questionnaire, the reason of iodine deficiency was unclear.

## Conclusion

In conclusion, we evaluated the iodine status and thyroid function of 1,264 women during pregnancy seven years after the modification of the USI standards. Our results revealed widespread iodine deficiency at any stage of pregnancy among women who did not take iodine supplements in Jiangsu Province. The follow-up study on euthyroid women negative for antithyroid Ab (TSH value of 2.5–3.55) confirmed that moderate-to-severe iodine deficiency (UIC lower than 100 μg/L) in the 1st trimester of pregnancy is associated with a significantly higher risk of SCH in subsequent stages of pregnancy. Our study is the first to report the association between iodine deficiency and the increased risks of SCH on the basis of two criteria. Our results highlight the need for periodic monitoring of iodine, especially in early pregnancy in Jiangsu Province, and present a basis for reducing the prevalence of SCH in the early stages of pregnancy.

## Data Availability Statement

The original contributions presented in the study are included in the article/Supplementary Material, further inquiries can be directed to the corresponding author/s.

## Ethics Statement

The studies involving human participants were reviewed and approved by Medical Ethics Committee of the Affiliated Hospital of Jiangsu University. The patients/participants provided their written informed consent to participate in this study.

## Author Contributions

TW conceived and designed the study. TW, YaL, XC, YuL, TZ, and SX collected the data. TW and QK analyzed the data. TW and LY drafted the manuscript. All authors reviewed the final manuscript. All authors contributed to the article and approved the submitted version.

## Funding

This work was supported in part by grants from Jiangsu Maternal and Child Health Research Project (F202101), Jiangsu Preventive Medicine Project (Y2018111), and the Foundation of Health Department, Jiangsu Province (X201424).

## Conflict of Interest

The authors declare that the research was conducted in the absence of any commercial or financial relationships that could be construed as a potential conflict of interest.

## Publisher's Note

All claims expressed in this article are solely those of the authors and do not necessarily represent those of their affiliated organizations, or those of the publisher, the editors and the reviewers. Any product that may be evaluated in this article, or claim that may be made by its manufacturer, is not guaranteed or endorsed by the publisher.
