# Supplementary material for: Effect of Moderate-to-Severe Iodine Deficiency in Early Pregnancy on Subclinical Hypothyroidism: A Longitudinal Study in an Iodine-Sufficient Region in China
Source: Front Nutr. 2022 Apr 1;9:839651. doi: 10.3389/fnut.2022.839651 (PMC9011046; doi:10.3389/fnut.2022.839651)
Supplement: Supplementary file 1 [file Image_1.pdf]

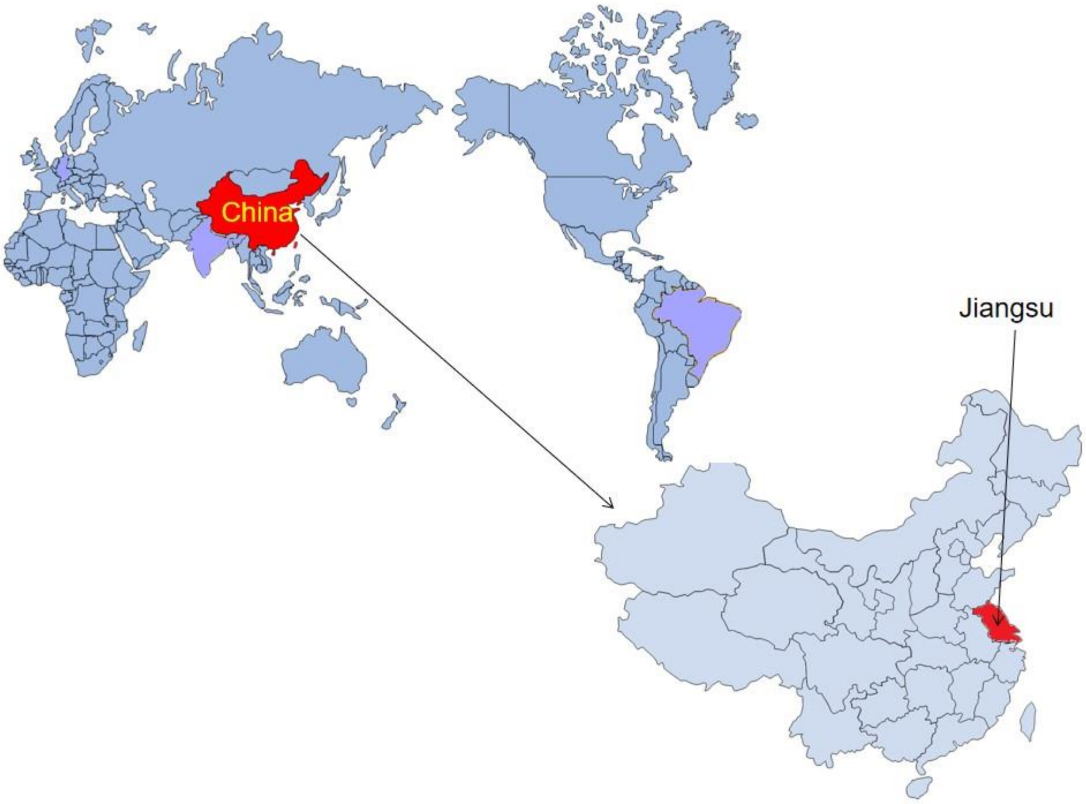

Supplementary Figure 1. Area map for the sampling territory. The sampling territory of this study is Jiangsu Province, China.
